# Mycosis fungoides unmasked by tralokinumab treatment for atopic dermatitis

**DOI:** 10.1016/j.jdcr.2025.03.030

**Published:** 2025-04-17

**Authors:** Sanah Basrai, Alexander R. Gomez-Lara, Dante Dahabreh, Sebastien De Feraudy, Eunice Y. Tsai, Patrick E. McCleskey

**Affiliations:** a Kaiser Permanente Bernard J Tyson School of Medicine, Pasadena, California; b Department of Internal Medicine, The Permanente Medical Group, Oakland, California; c Department of Pathology, The Permanente Medical Group, Oakland, California; d Department of Dermatology, The Permanente Medical Group, Union City, California; e Department of Dermatology, The Permanente Medical Group, Oakland, California; f Department of Clinical Science, Kaiser Permanente Bernard J Tyson School of Medicine, Pasadena, California

**Keywords:** atopic dermatitis, cutaneous T-cell lymphoma, mycosis fungoides, tralokinumab

## Introduction

Atopic dermatitis (AD) is a chronic skin condition characterized by erythematous plaques, pruritus, and TH2-dominated inflammation. Dupilumab, the first biologic approved by the U.S. Food and Drug Administration in 2017 for moderate-to-severe AD, blocks interleukin 4 (IL-4) and IL-13 signaling by binding to interleukin 4 receptor α-antagonist, the shared receptor for both cytokines.^1^ Tralokinumab, approved in 2021, is a human IgG4 monoclonal antibody targeting only IL-13, blocking its receptor interaction and neutralizing downstream signaling, while allowing normal IL-4 signaling to continue.^2^

Dupilumab has been associated with cutaneous T-cell lymphoma (CTCL), with theories suggesting that immune pathway alterations due to drug targeting may unmask clonal T-cell proliferation, or alternatively that CTCL was initially misdiagnosed as AD.^3^ We report a rapidly progressive and fatal case of tralokinumab-associated mycosis fungoides (MF) with severe erosive changes.

## Case report

A 73-year-old HIV-negative female with a history of AD and asthma since childhood presented to the dermatology department in January 2020 for a keratoacanthoma and AD. Emollients, topical steroids, and topical calcineurin inhibitors failed to control her symptoms. Narrowband UVB therapy was administered in the clinic for 2 years. She was reluctant to use methotrexate or mycophenolate mofetil, and dupilumab was cost prohibitive.

Following 2 years of nbUVB therapy, a punch biopsy of the right thigh revealed subacute spongiotic dermatitis with eosinophils, consistent with a diagnosis of subacute eczematous dermatitis. Direct immunofluorescence studies were negative. She maintained with tacrolimus, topical steroids, and home nbUVB therapy, with mild improved itch.

Five months postbiopsy, she had focal patches of hair loss involving the scalp and eyebrows, with continued itch. By 8 months postbiopsy, there was worsening itch and was prescribed tralokinumab (600 mg loading dose, then 300 mg every 2 weeks). She started the therapy after a month of administrative delay in receiving the tralokinumab, and the patient discontinued home nbUVB therapy. Eleven months postbiopsy, she returned with worsening erosions, dysesthesia, and intractable itch, which was treated with cefadroxil for secondary impetiginization.

**Fig 1:**
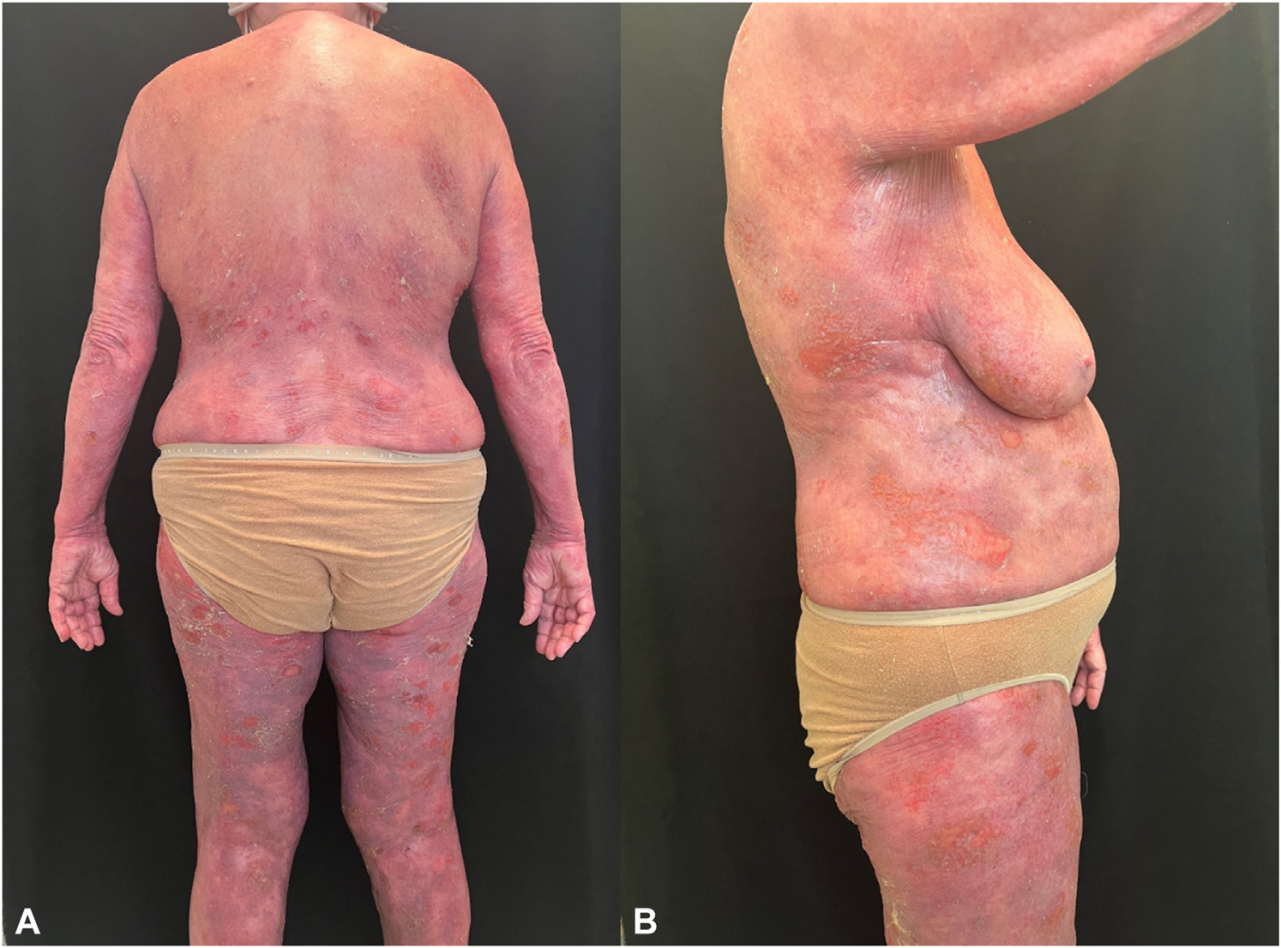
**A** and **B,** Widespread erythema and desquamating scale with eroded atrophic patches over the back, legs, and abdomen 2 days prior to hospital admission.

**Fig 2:**
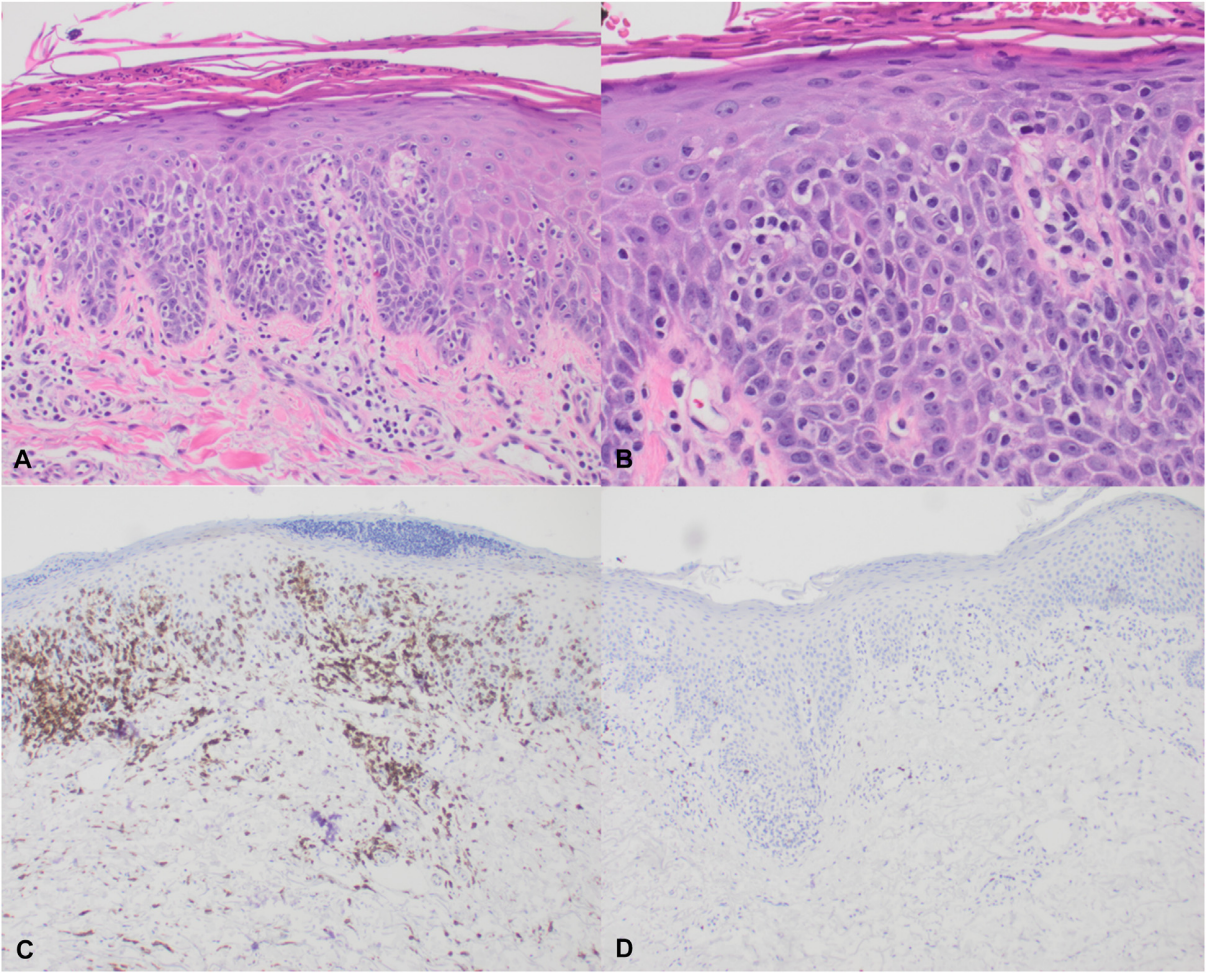
Histopathology slides of the August 2024 punch biopsy. **A** and **B,** H&E stains show a characteristic atypical epidermotropic infiltrate of small to medium size lymphocytes with irregular, hyperchromatic, cerebriform nuclei (**A**, 200×; **B**, 400×). **C,** Immunohistochemical labeling with CD4 highlights the atypical epidermotropic lymphocytes (200×). **D,** Immunohistochemical labeling with CD8 demonstrates a lack of positivity in the atypical epidermotropic lymphocytes (200×). *H&E*, Hematoxylin and eosin.

A year following the initial biopsy, and after 4 doses of tralokinumab, the patient's condition worsened to erythroderma with excoriated erosions on arms, legs, back, neck, and buttocks, without lymphadenopathy (Fig 1). Biopsy of the right thigh and left arm revealed psoriasiform hyperplasia with numerous intraepidermal CD4+ lymphocytes with extensive epidermotropism, dermal eosinophils, and spongiosis (Fig 2). T-cell gene rearrangement (TCR) studies were positive for the skin sample. Tralokinumab was discontinued. Computed tomography scan of chest/abdomen/pelvis was negative. WBC was 9700/uL, but differential was not done that day. Flow cytometry showed CD3 cells 235/uL, CD4:CD8 ratio 1.51, CD4+ CD7^‒^ T cells 16% of CD4+ T cells, CD4+ CD26^‒^ T cells 43% of CD4+ T cells. TCR was negative due to inadequate genetic material. She did not meet the criteria for Sezary syndrome; the diagnosis of erosive MF was made. TCR on the original biopsy was requested despite the results not demonstrating features of MF on hematoxylin and eosin stain. The TCR report was positive with similar clones as the 2 diagnostic biopsies for MF. Treatment with methotrexate and prednisone was initiated, and she restarted home nbUVB therapy.

Two months later, she reported intractable burning pain and itching and was started on cefadroxil, gabapentin, and wet wraps with triamcinolone ointment. She presented to urgent care and was admitted to the hospital for pain control, dehydration, hypokalemia, and skin care management. Blood cultures grew methicillin-susceptible *Staphylococcus aureus* and later *Serratia marcescens*. Her hospital course was complicated by leukopenia, anemia, sedation, and bacteremia. Bone marrow biopsy was offered but declined, and blood products were transfused. Our oncology team did not think she could tolerate any other CTCL treatments in the setting of septicemia and low blood counts. Despite multiple antibiotics and escalation to intensive care, she succumbed to sepsis and passed away after a 3-week hospital course.

## Discussion

We describe a case of tralokinumab unmasking MF in a patient with lifelong AD. The clinical scenario was marked by an acute worsening in the patient's condition while on tralokinumab, which revealed an undiagnosed MF masquerading as worsening AD. This was confirmed when a TCR analysis performed on the original skin biopsy demonstrated clonality matching later biopsies that confirmed MF.

Advanced MF is associated with a Th2-dominated microenvironment characterized by the upregulation of IL-13 receptors. IL-13 activates the transcription factor signal transducer and activator of transcription 6, stimulating T cells and contributing to CTCL progression. This observation promoted the hypothesis that IL-13 blockers such as dupilumab and tralokinumab could be beneficial for treating MF.^4^

However, since 2019, several reports have documented MF diagnosis following dupilumab administration. The leading theory suggests that dupilumab may unmask underlying MF due to several factors: (1) lack of improvement on a biologic prompts further investigation for rare conditions like CTCL; (2) early-stage MF mimics AD on biopsy and in symptomatology; (3) CTCL can arise in the setting of prolonged inflammation, such as AD, and dupilumab may clear the initial disease pathology, making CTCL more evident.^4^, ^5^, ^6^ Supporting this theory, an analysis of large claims data conducted by Neubauer et al^6^ found no increased incidence of CTCL with dupilumab compared to prednisone, cyclosporine, or methotrexate. The authors suggest that dupilumab does not heighten CTCL risk but may instead unmask MF that had yet to be diagnosed—a supposition that likely applies to tralokinumab in this case.^6^ The diagnosis of MF may require multiple biopsies, and the diagnostic accuracy is improved if the same clone is found in 2 separate skin specimens.^7^

When considering anti-IL-13 therapy for patients with atypical or worsening AD, our case suggests that 2 skin biopsies from more than 1 skin site may be warranted to exclude MF. The clinician submitting the biopsy must communicate the suspected MF and potential anti-IL-13 therapy to the dermatopathologist to ensure appropriate immunohistochemical and TCR studies when hematoxylin and eosin histopathology slides do not meet the criteria for MF. Before initiating anti-IL-13 therapy, caution is recommended if any clonality is seen on TCR. Clear communication between the dermatologist and the dermatopathologist is critical when considering anti-IL-13 therapy in progressive AD.

## Conflicts of interest

None disclosed.

## References

[1] Seegräber M., Srour J., Walter A., Knop M., Wollenberg A. (2018). Dupilumab for treatment of atopic dermatitis. Expert Rev Clin Pharmacol.

[2] Guttman-Yassky E., Kabashima K., Staumont-Salle D. (2024). Targeting IL-13 with tralokinumab normalizes type 2 inflammation in atopic dermatitis both early and at 2 years. Allergy.

[3] Park A., Wong L., Lang A., Kraus C., Anderson N., Elsensohn A. (2023). Cutaneous T-cell lymphoma following dupilumab use: a systematic review. Int J Dermatol.

[4] Mazzetto R., Miceli P., Tartaglia J., Ciolfi C., Sernicola A., Alaibac M. (2024). Role of IL-4 and IL-13 in cutaneous T cell lymphoma. Life (Basel).

[5] Toker M., Srivastava P., Amin B., Wu B. (2023). Did dupilumab unmask smoldering mycosis fungoides?. JAAD Case Rep.

[6] Neubauer Z.J.K., Brunner P.M., Geskin L.J., Guttman E., Lipner S.R. (2024). Decoupling the association of dupilumab with cutaneous T-cell lymphoma. J Am Acad Dermatol.

[7] Thurber S.E., Zhang B., Kim Y.H., Schrijver I., Zehnder J., Kohler S. (2007). T-cell clonality analysis in biopsy specimens from two different skin sites shows high specificity in the diagnosis of patients with suggested mycosis fungoides. J Am Acad Dermatol.

